# Nanostructured silica spin–orbit optics for modal vortex beam shaping

**DOI:** 10.1515/nanoph-2021-0579

**Published:** 2021-12-15

**Authors:** Delphine Coursault, Etienne Brasselet

**Affiliations:** Univ. Bordeaux, CNRS, LOMA, UMR 5798, Talence, France

**Keywords:** beam shaping, geometric phase, Laguerre Gauss, optical mode; optical vortex, polarization

## Abstract

Modality is a generic concept of wave-optics at the basis of optical information and communications. One of the challenges of photonics technologies based on optical orbital angular momentum consists in the production of a modal content for both the azimuthal and radial degrees of freedom. This basically requires shaping the complex amplitude of an incident light beam, which is usually made up from adaptive spatial light modulators or bespoke devices. Here, we report on the experimental attempt of a recent theoretical proposal [*Opt. Lett.* **42**, 1966 (2017)] toward the production of various optical vortex modes of the Laguerre–Gaussian type relying on the spin–orbit interaction of light. This is done in the visible domain from optical elements made out of silica glass. The idea consists in exploiting the combined effects of azimuthally-varying geometric phase with that of radially-varying propagation features. The proposed approach can be readily extended to any wavelength as well as to other families of optical modes, although some dynamic phase problems remain to be solved to make it a turnkey technology.

## 1 Introduction

The concept of modes is ubiquitous to optics as the traditional use of Gaussian beam recalls. Still, in paraxial wave optics, the customary Gaussian beam only represents the fundamental mode of many kinds of infinite dimensional complete orthogonal basis that are exact solutions of the scalar paraxial Helmholtz equation. For instance, we mention the Hermite–Gauss basis in Cartesian coordinates, the Laguerre–Gauss basis in cylindrical coordinates and the Ince–Gauss basis in elliptical coordinates. Among the latter basis, the Laguerre-Gauss one became a prototypical basis in the field of structured light and its applications [1]. This has happened after its constitutive elements have been recognized to carry an integer multiple of *ℏ* of optical orbital angular momentum per photon [2] associated with their field amplitude proportional to exp(*ilϕ*), where *l* is an integer and *ϕ* is the azimuthal angle in a plane transverse to the beam propagation direction. Since then, after three decades of advances, a plethora of so-called optical vortex generator devices imparting to an incident field a pure phase transmittance or reflectance proportional to exp(*ilϕ*) have been realized [3]. Such optical elements can be classified depending on the physical nature of the involved phase: *dynamic* when associated with the propagation of light and *geometric* when associated with the structure of the material. Still, none of them generates Laguerre-Gauss beams, which are associated with two independent spatial degrees of freedom, which transpires from their classification by two indices, l∈Z and p∈N . Omitting the usual free-space propagation factor exp(−*iωt* + *ik*_0_ *z*) the electric field of the mode (*l*, *p*) propagating toward *z* > 0 is expressed in the cylindrical coordinates (*r*, *ϕ*, *z*) as [4]

(1) $$\begin{array}{ll} E_{l,p}\left(r,\phi ,z;w_{0}\right) & \propto \frac{w_{0}}{w\left(z\right)}{\left[\frac{r}{w\left(z\right)}\right]}^{\;|l|}\;\;L_{p}^{\left|l\right|}\;\left(\frac{2r^{2}}{w{\left(z\right)}^{2}}\right)\\ & \;\times \exp\;\left[-\frac{r^{2}}{w{\left(z\right)}^{2}}\right]\exp\;\left\{i\left[\frac{k_{0}r^{2}z}{2\left(z^{2}+z_{0}^{2}\right)}\right.\right.\\ & \left.\left.\;+l\phi -\left(2p+|l|+1\right)\;\arctan\;\left(\frac{z}{z_{0}}\right)\right]\right\}, \end{array}$$

where $L_{p}^{\left|l\right|}\left(x\right)=\sum_{m=0}^{p}\frac{\left(|l|+p\right)!}{\left(|l|+m\right)!\left(p-m\right)!m!}{\left(-x\right)}^{m}$ with *x* = 2*r* ^2^/*w*(*z*)^2^ refers to the associated Laguerre polynomials, *w*_0_ is the beam waist radius, $z_{0}=k_{0}w_{0}^{2}/2$ and $w\left(z\right)=w_{0}\sqrt{1+{\left(z/z_{0}\right)}^{2}}$ . Generating a mode (*l*, *p*) from a Gaussian beam thus requires both phase and amplitude shaping. This can be done in various ways, for instance by using a phase-only spatial light modulator, where the basic trick to modulate the amplitude is to induce on-axis transmission losses by redirecting light to either zeroth or higher diffraction orders, see [5] for a recent survey of modal beam shaping.

Some years ago, a polarization-based approach that does not require energy-redirection strategy – but instead exploits the two degrees of freedom of a polarization ellipse (orientation and ellipticity) – has been theoretically proposed [6]. The basic idea is grasped from how a standard uniform birefringent retarder lying in the (*x*, *y*) plane modifies the polarization state of a normally incident circularly polarized light with wavelength *λ*. Introducing the Jones vectors $c_{\sigma }=\left(x+i\sigma y\right)/\sqrt{2}$ for circular polarization states with helicity *σ* = ±1, we get

(2) $$c_{\sigma }\underset{\psi ,\delta }{\to }\cos\left(\pi \delta /\lambda \right)\;c_{\sigma }+i\sin\left(\pi \delta /\lambda \right)\;{\text{e}}^{\text{i}2\sigma \psi }c_{-\sigma },$$

where *δ* is the retardance and *ψ* is the in-plane polar orientation angle of the slow-axis of a slab made of a uniform uniaxial medium. Post-selecting the **c**_−*σ*_ polarization component thus gives amplitude and phase shaping from retardance and orientation, respectively.

Very recently, the light beam shaping capabilities of the above approach have been the subject of further theoretical analysis [7] and have also been theoretically rediscovered in the context of dielectric optical metasurfaces [8]. From an experimental point of view, an approximate experimental attempt to generate Laguerre–Gauss modes with *p* = 0 has been reported in the limit of small retardance from nanostructured silica glass; however, it was at the expense of efficiency and technologically unavoidable retardance bias *δ*_bias_ ∼ 60 nm at that time [9]. More recently, an attempt relying on the fabrication of metallic metasurfaces has been reported [10], yet without experimental assessment of the expected propagation-invariant features of the generated modal beams. To date, a thorough experimental demonstration of dissipation-free doubly-inhomogeneous modal optical elements thus remains elusive and the present work aims at filling this gap in the context of optical vortex modes.

Here, owing to recent developments of ultrafast laser nanostructuring of glasses that allow considering the realization of low-loss and bias-free spatially modulated retardance [11], we experimentally report on optimized Laguerre–Gaussian beam shapers made of silica glass. We first present the case of modes with fundamental radial order *p* = 0 modes and various *l*, then we extend the results to high-order radial modes *p* ≠ 0 at fixed *l* = 1. Finally, the results are critically analyzed and we provide a short survey of material, technological and photonic prospects in the context of modal beam shaping.

## 2 Design and fabrication

From a general point of view, let us consider a *σ*-polarized incident Gaussian mode with beam waist radius *w*_in_ impinging at normal incidence on the modal shaper placed at *z* = 0. The incident field in the plane of the sample is thus described according to Eq. (1) by taking (*w*_0_, *l*, *p*) = (*w*_in_, 0, 0). According to the method described in [6], we use the following retardance and slow axis orientation in-plane designs for modal optical elements with *p* = 0:

(3) $$\delta_{l,0}\left(r\right)=\frac{\lambda }{\pi }\arcsin\left\{\;{\left(\frac{r\sqrt{2}}{w_{\text{in}}}\right)}^{\;\;|l|}\;\;\;\exp\;\left[|l|\;\left(\frac{1}{2}-\frac{r^{2}}{w_{\text{in}}^{2}}\right)\right]\right\}$$

(4) $$\psi_{l,0}\left(\phi \right)=\sigma l\phi /2.$$

This corresponds to an output beam waist radius $w_{\text{out}}=w_{\text{in}}/\sqrt{1+|l|}$ and maximal modal output power fraction *η*_*l*,0_ = |*l*|!e^|*l*|^/(1 + |*l*|)^1+|*l*|^ with respect to the incident beam power. In the following, the modal performances of the optical elements associated with the design given by Eqs. (3) and (4) are benchmarked with respect to standard optical vortex shapers having uniform retardance and azimuthally-varying slow axis orientation in the plane of the sample. Namely,

(5) $$\delta_{\text{standard}}=\lambda /2$$

(6) $$\psi_{\text{standard}}\left(\phi \right)=\sigma l\phi /2$$

The fabrication of that kind of standard optical elements using state of the art low-loss ultrafast laser nanostructuring of glass has been fully described in [11], though without analyzing the propagation properties of the generated optical vortex beams in the context of modality, the which is done in the present work. Finally, we recall that the sign of the azimuthal index of the generated mode can be switched by flipping the incident helicity (*σ* → −*σ*) while keeping the same optical element, hence preserving the basic feature of spin-controlled wavefront reversal of geometric phase optical elements as originally introduced by Bhandari [12]. Therefore, without lacking generality, we use *σ* = +1 from now on.

**Figure 1:**
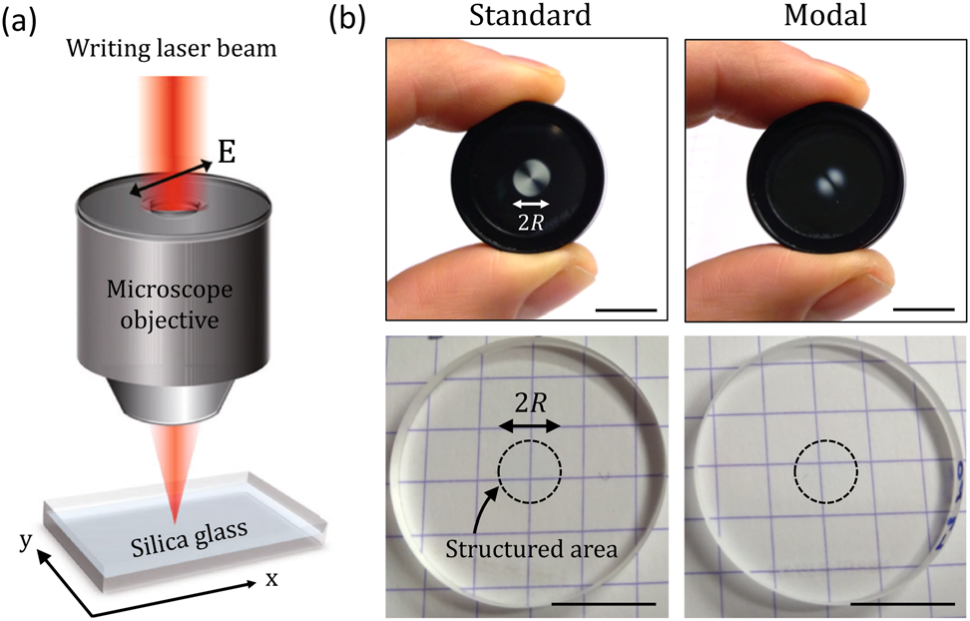
Main characteristics of the samples. (a) Sketch of the ultrafast laser writing of inside a slab of silica glass adapted from [11], where the double arrow refer to the orientation of the linearly polarized writing beam described by the electric field **E**. (b) Pictures of the standard [*l* = 1] and modal [(*l*, *p*) = (1, 0)] vortex beam shapers observed in ambient light conditions through crossed linear polarizers (upper panels) and when placed on the top of a sheet of graph paper with 5 × 5 mm grid (bottom panels). The optical vortex shapers have a disk-shaped footprint of diameter 2*R* = 6 mm and are embedded in a disk slab of diameter 1 inch and thickness 3 mm, typically at a 0.2 mm distance below the air/glass interface from which impinges the writing laser beam. Scale bar: 1 cm.

The space-variant birefringent properties of the fabricated optical elements (from Workshop of Photonics) results from light-induced structural changes at the nanoscale in the bulk of a slab of silica glass of type UVFS Corning 7980 according to the technique reported in [11], to which we refer the reader for experimental details. In brief, femtosecond laser operating at 1030 nm wavelength with tunable repetition rate and pulse duration is focused inside the glass slab using a low numerical aperture lens, as depicted in Figure 1a. The laser induced retardance and slow axis orientation are controlled by adjusting the pulse energy and density as well as the polarization azimuth of the writing beam to every location (*r*, *ϕ*) in the plane of the slab and several birefringent layers are stacked as needed to achieve the desired retardation value. Both standard and modal vortex shapers are fabricated to operate for *w*_in_ = 1.5 mm at *λ* = 532 nm and have a disk-shaped footprint of radius *R* = 3 mm. Pictures of the optical elements between crossed linear polarizers are shown in the upper part of Figure 1b, which allows grasping at a glance that standard vortex shapers have uniform retardance (i.e., constant intensity along *r*) and inhomogeneous slow axis orientation (i.e., periodic intensity changes along *ϕ*) while modal vortex shapers are doubly-inhomogeneous. In addition, the bright field pictures shown in the bottom part of Figure 1b qualitatively illustrate that both absorption and scattering losses due to the nanostructuring are negligible as the grid lines of the graph paper on the top of which are placed the optical elements are visually unaltered.

## 3 Structural characterization

**Figure 2:**
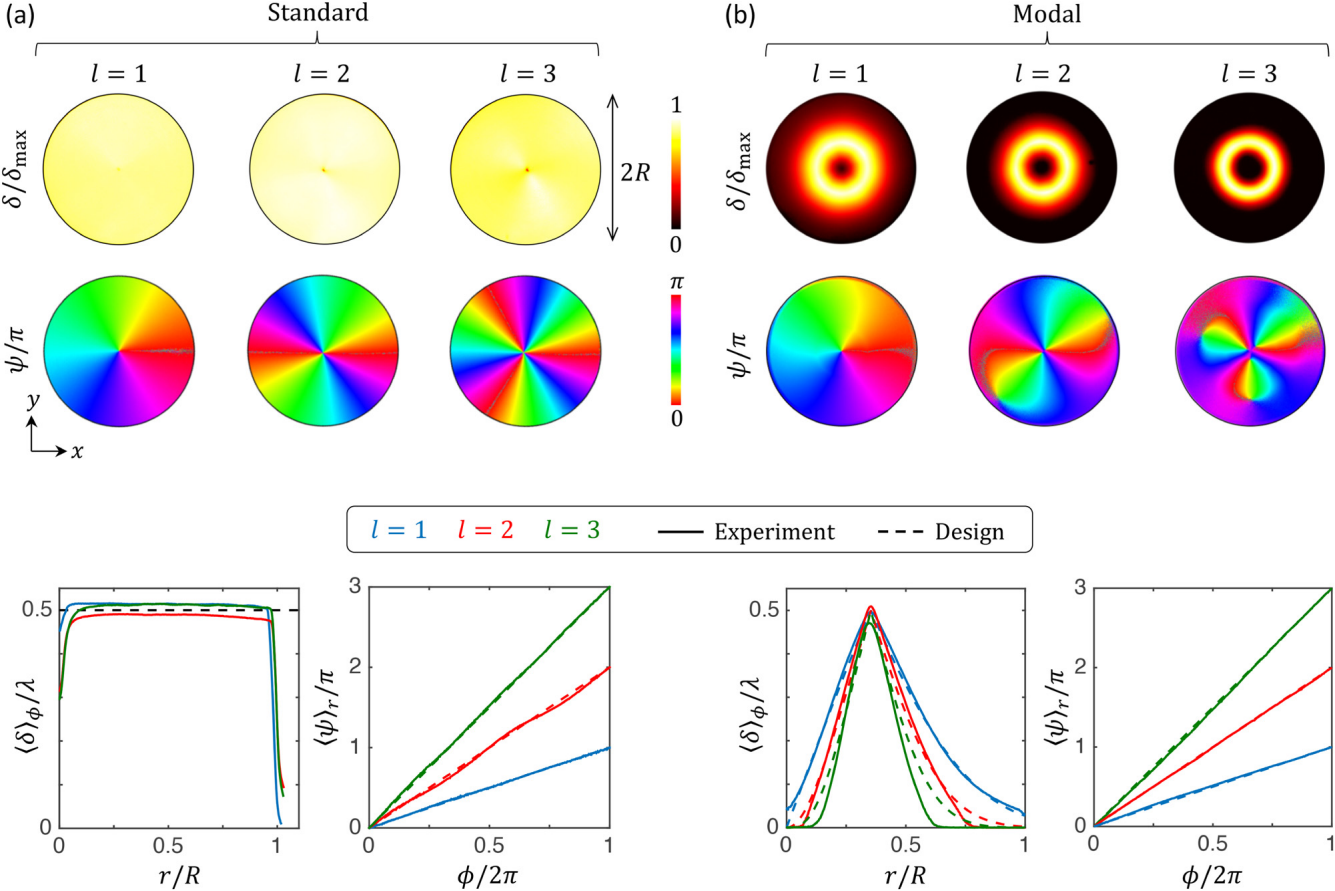
Structural polarimetric characterization of the samples. (a) and (b) Experimental spatial distributions of the retardance *δ* and azimuth *ψ* retrieved by polarimetric imaging of the nanostructured area of diameter 2*R* for standard and modal (*p* = 0) vortex beam shapers, respectively, for *l* = 1 (red), *l* = 2 (green) and *l* = 3 (blue). Upper part: normalized maps *δ*/*δ*_max_ and *ψ*/*π* in the (*x*, *y*) planes, where *δ*_max_ = max[*δ*(*x*, *y*)], for *l* = (1, 2, 3). Bottom part: radial profiles of the reduced retardance ⟨*δ*⟩_*ϕ*_/*λ* and azimuthal profiles of the reduced azimuth ⟨*ψ*⟩_*r*_/*π*, where ⟨…⟩_*ϕ*,*r*_ respectively refers to azimuth-average over the range 0 < *ϕ* < 2*π* and radial-average over the range 0 < *r* < *R* (standard case) and 0.2*R* < *r* < 0.45*R* (modal case). Solid lines: experimental data. Dashed lines: analytical designs given by {*δ*_standard_, *ψ*_standard_} (a) and {*δ*_*l*,0_, *ψ*_*l*,0_} (b).

The structural characterization of the modal vortex plates is made via polarimetric imaging of the samples. The principle of this technique consists of determining the output Stokes parameters (*S*_0_, *S*_1_, *S*_2_, *S*_3_) [13] on the entire surface of the optical elements. Samples are illuminated at normal incidence by a collimated circularly polarized probe light field with helicity *σ*_probe_ that is obtained from a halogen lamp spectrally filtered at *λ*_probe_ = 633 nm. Noting that the maximal expected retardance is *λ*/2, the retardance and the slow axis orientation are respectively evaluated from the relationships *δ* = (*λ*_probe_/2*π*) arccos(*σ*_probe_ *S*_3_/*S*_0_) and *ψ* = Ψ − *σ*_probe_ *π*/4, where *Ψ* = (1/2) arctan(*S*_2_/*S*_1_) is the azimuth angle of the polarization ellipse of the output probe light field. Obtained maps for *δ* and *ψ* over the disk-shaped footprint of the nanostructured glass are shown in the upper part of Figure 2. These maps allow the quantitative assessment of the radial retardance profiles as well as the azimuthal azimuth profiles, see the bottom part of Figure 2. The comparison with the analytical design allows appreciating the quality of the realized standard and modal vortex beam shapers, which calls for a few comments in both cases as detailed hereafter.

For the standard elements, we observe a non-zero retardance for *r* > *R* while the laser nanostructuring is restricted to *r* < *R*. This peripheral retardance is photoelastic in nature and results from mechanical stresses in the vicinity of the structural discontinuity at *r* = *R*. Also, the observed on-axis retardance typically drops for *r* < *R*/20 is concomitant to the laser writing protocol when a singular spatial distribution for the nanogratings wavevector orientation is sought after. In fact, when dealing with subwavelength structured optical vortex shapers, the central area is customarily left unstructured whatever the used technology, especially for large values of *l* [14]. These limitations have however limited effects as the incident beam waist is large enough to prevent that a substantial fraction of the incident power (in practice of the order of a percent) is affected by on-axis lack of resolution. In addition, having chosen *w*_in_ = *R*/2 ensures that virtually no optical power falls outside the footprint of the element.

For the modal elements, we observe that the larger the azimuthal index *l*, the more the retardance profile departs from the design. This is an experimental limitation that mainly comes from two issues. First, the retardance value does not vary linearly with respect to the pulse energy. Second, the retardance differs for different polarization orientation of the writing beam with respect to the writing beam displacement direction (so-called ‘quill writing’ effect [15, 16]). In addition, the abrupt variation at $r=w_{\text{in}}/\sqrt{2}$ for the designed retardance suffers from smoothening, as one would expect from the finite resolution of the laser writing process. Moreover, the larger the azimuthal index *l*, the more the measured maps for the slow axis orientation appear to deviate from the *r*-independent maps expected from Eq. (4). Noteworthy, this can be explained by the fact that near-zero retardance leads to negligible changes of the incident circular polarization state. Therefore, recalling that both *S*_1_ and *S*_2_ are nearly zero for nearly circular polarization states, the non-ideal nature of the used polarization optical elements leads to noisy determination of the ratio *S*_2_/*S*_1_. So does the determination of *ψ* in the central part and at the periphery of the optical elements, where the retardance is near-zero. As a result, flower-like *ψ*-maps are observed and the effect is more pronounced as *l* increases because the size of the region of small retardance at the center and at the periphery correspondingly increases. Said differently, care should be exercised before stating that the mismatch with respect to the expectations from the design is due to imperfections of the fabrication process.

## 4 Beam propagation analysis

**Figure 3:**
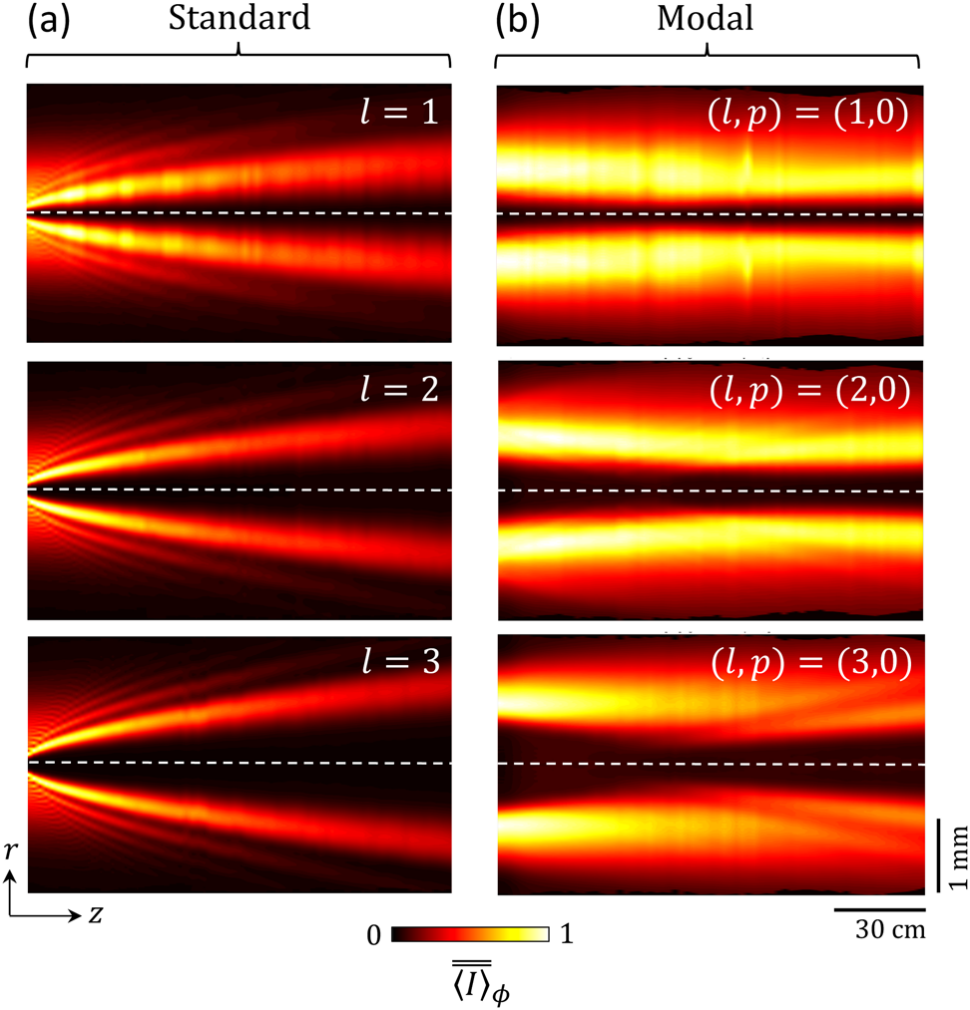
Beam propagation. Experimental normalized azimuth-average intensity distribution in the (*z*,*r*) plane for standard (a) and modal (b) vortex beam shapers with *l* = (1, 2, 3). We define ${\bar{\bar{\left\langle I\right\rangle }}}_{\phi }={\left\langle I\right\rangle }_{\phi }/\underset{r,z}{\max}\left[{\left\langle I\right\rangle }_{\phi }\right]$ . The experimental data is recorded directly by a camera displaced along the *z* axis, the which is indicated here as the dashed line, without use of a relay-lens system and post-selecting the relevant circularly polarized component by placing a circular polarizer between the optical element and the camera. The leftmost part of each panel refers to the smallest distance from the sample at which direct imaging can be achieved, namely, *z*_min_ = 4 cm. We note that, in the modal case, the designed output waist values *w*_out_ = (1.06, 0.87, 0.75) mm for *l* = (1, 2, 3) are associated to the Rayleigh distances *z*_0_ = (6.64, 4.43, 3.32) m, which explains the observed trend of an increase of the beam divergence as *l* increases.

**Figure 4:**
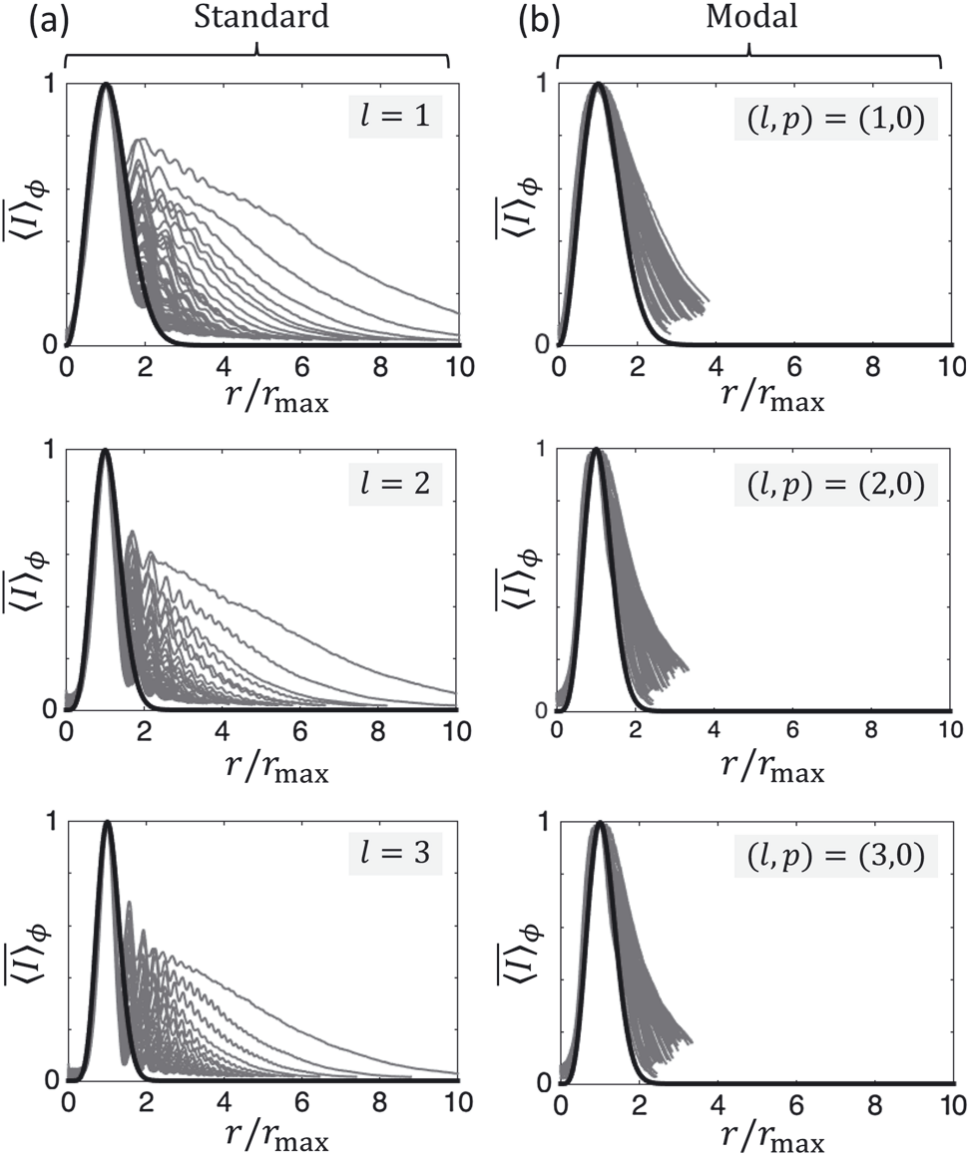
Reduced azimuth-average radial intensity distribution that corresponds to the data shown in Figure 3. We define ${\bar{\left\langle I\right\rangle }}_{\phi }={\left\langle I\right\rangle }_{\phi }/\underset{r}{\max}\left[{\left\langle I\right\rangle }_{\phi }\right]$ where *r*_max_ is the distance from the axis at which ⟨*I*⟩_*ϕ*_ is maximum at a fixed *z*, for standard (a) and modal (b) vortex beam shapers with *l* = (1, 2, 3). Gray curves: experimental data. Black curves: propagation-invariant reduced Laguerre–Gauss profiles ${\left(\rho \sqrt{2/|l|}\right)}^{2|l|}\exp\left(1-2\rho^{2}\right)$ where *ρ* = *r*/*r*_max_.

The beam shaping propagation features of the fabricated standard and modal vortex shapers are retrieved by recording the transverse intensity profiles as a function of the propagation distance up to *z* = 1.5 m. The results are presented in Figure 3, which highlights distinct propagative behaviors for the standard approach and the modal approach. In the standard case, the incident Gaussian beam strongly diffracts on the large gradients of the material structure in the vicinity of the central part, which feeds a substantial amount of the incident power into higher-order radial modes with *p* ≠ 0. Quantitatively, the calculated fraction of the incident power transformed into the mode (*l*, 0) equals $\frac{2^{\left|l\right|}|l{|}^{2}}{1+|l|}{\left(\frac{1+|l|}{2+|l|}\right)}^{2+|l|}\frac{\Gamma^{2}\left(|l|/2\right)}{\Gamma \left(1+|l|\right)}≃\left(0.93,0.84,0.77,\ldots \right)$ for the standard design with *l* = (1, 2, 3, …), where Γ(⋅) is the gamma function [17]. In contrast, we observe that the ideally vanishing coupling to high-order radial modes is strongly reduced for the modal design, as reported in Figure 3. The ensuing shape invariance for the transverse intensity profile, which is a remarkable property of Laguerre–Gaussian beams, is assessed in Figure 4. There, the reduced azimuth-average radial intensity profiles are shown for a set of over 70 values for the propagation distance *z* (gray curves) and compared to the corresponding analytical Laguerre–Gauss reduced profile (black curves). The enhanced propagation invariance of the modal experimental attempt is clearly visible.

**Figure 5:**
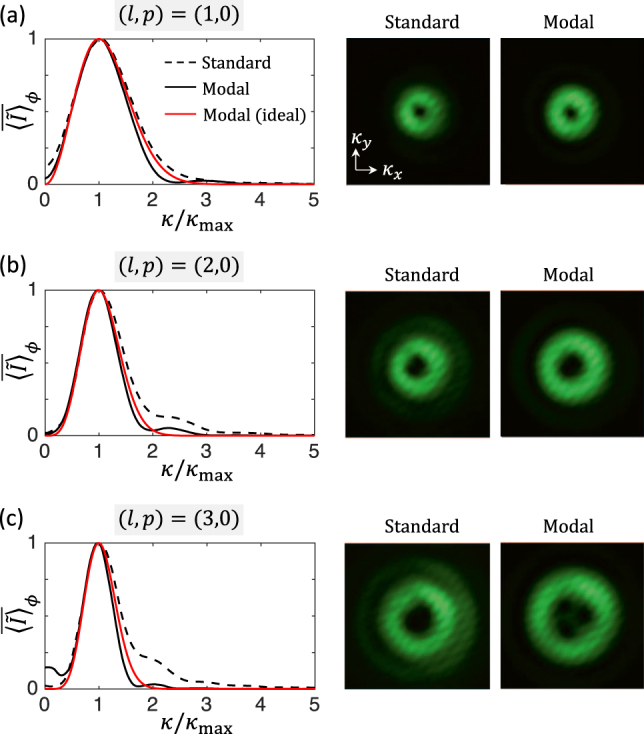
Reduced azimuth-average far field intensity distribution for standard and modal (*p* = 0) vortex beam shapers with *l* = (1, 2, 3). We define ${\bar{\left\langle \tilde{I}\right\rangle }}_{\phi }={\left\langle \tilde{I}\right\rangle }_{\phi }/\underset{\kappa }{\max}\left[{\left\langle \tilde{I}\right\rangle }_{\phi }\right]$ where $\kappa ={\left(\kappa_{x}^{2}+\kappa_{y}^{2}\right)}^{1/2}$ is the magnitude of the spatial frequencies in the transverse plane and *κ*_max_ is the spatial frequency magnitude at which ${\left\langle \tilde{I}\right\rangle }_{\phi }$ is maximum. The data is recorded by placing a lens with focal length *f* = 50 cm in *f*–*f* configuration between the sample and the camera. The experimental far field intensity distributions are shown on the right part of the figure and are all displayed using identical scale.

Still we notice that the far field intensity patterns qualitatively look like Laguerre–Gauss modes whatever the standard or modal nature of the optical element. This is illustrated in Figure 5 that displays the azimuth average radial intensity profiles as well as the corresponding far field transverse intensity distribution both for standard and modal vortex beam shapers. This explains why standard vortex beam shapers made of helical phase masks are often, yet incorrectly, said to produce Laguerre–Gauss fields *E*_*l*,0_ despite several works have already pinpointed this issue, see for instance [18, 19]. Finally, from a close look at the inner part of the doughnut intensity distribution for *l* > 1 in Figure 5 one may notice that high-order optical vortices split into unit charge vortices. Besides the fact that this can result from structuring imperfections, it is worth recalling the non-generic nature of high-order optical phase singularities that makes them unstable to perturbations (for instance a small coherent background) as observed since the early days of optical vortex generation [20].

## 5 Extension to high-order radial modes

Our approach is then extended to high-order radial modes by using the following retardance and azimuth designs [6]:

(7) $$\delta_{l,p}\left(r\right)=\frac{\lambda }{\pi }\arcsin\;\left\{\;\frac{|E_{l,p}\left(r,0,0;\zeta_{l,p}w_{\text{in}}\right)|e^{r^{2}\;/w_{\text{in}}^{2}}}{\max\;\left[|E_{l,p}\left(r,0,0;\zeta_{l,p}w_{\text{in}}\right)|e^{r^{2}\;/w_{\text{in}}^{2}}\right]}\;\right\}$$

(8) $$\psi_{l,p}\left(\phi \right)=\sigma l\phi /2-\frac{\pi }{4}\left\{1-\mathrm{sign}\;\left[L_{p}^{\left|l\right|}\left(2r^{2}/w_{\text{in}}^{2}\right)\right]\right\}$$

In the above equations, the parameter 0 < *ζ*_*l*,*p*_ < 1 that define the output beam waist radius *w*_out_ = *ζ*_*l*,*p*_ *w*_in_ is chosen in order to get maximal modal output power fraction. In contrast to the case *p* = 0, *ζ*_*l*,*p*≠0_ has to be determined numerically. Here, we restrict our investigations to *l* = 1 and *p* = (1, 2) which are associated with *ζ*_1,*p*_ = (0.44, 0.38).

**Figure 6:**
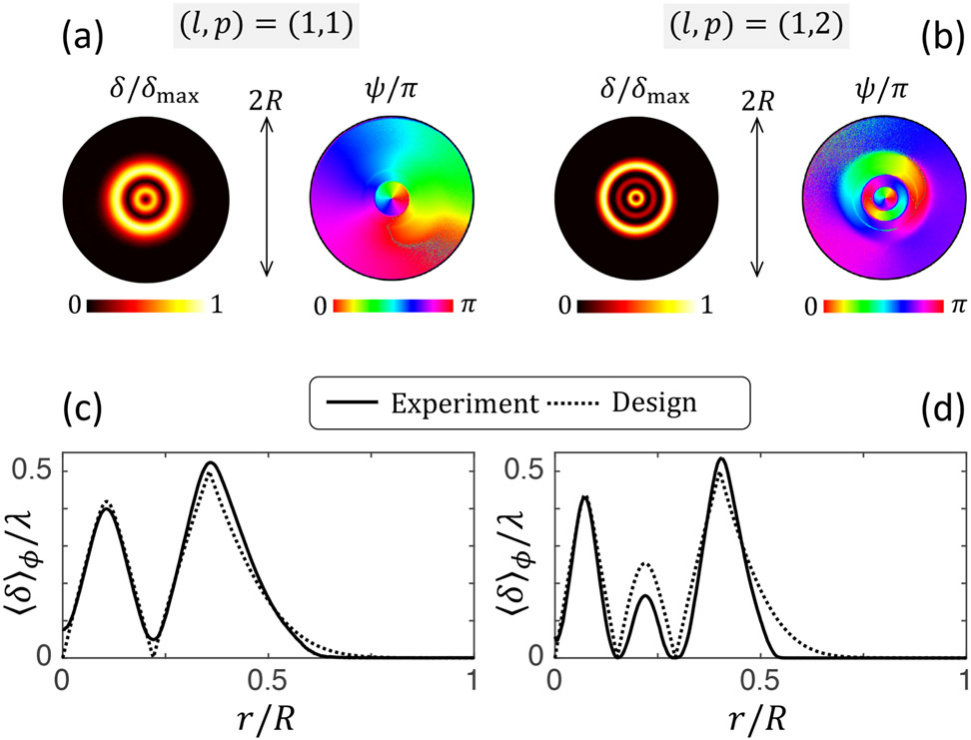
Experimental structural characterization of the modal vortex shapers with (*l*, *p*) = (1, 1) and (*l*, *p*) = (1, 2). (a) and (b) Spatial distribution of the retardance *δ* and azimuth *ψ* spatial distributions retrieved by optical polarimetric means. (c) and (d) Radial profiles of the reduced retardance extracted from the latter maps. The notations are identical to that of Figure 2.

**Figure 7:**
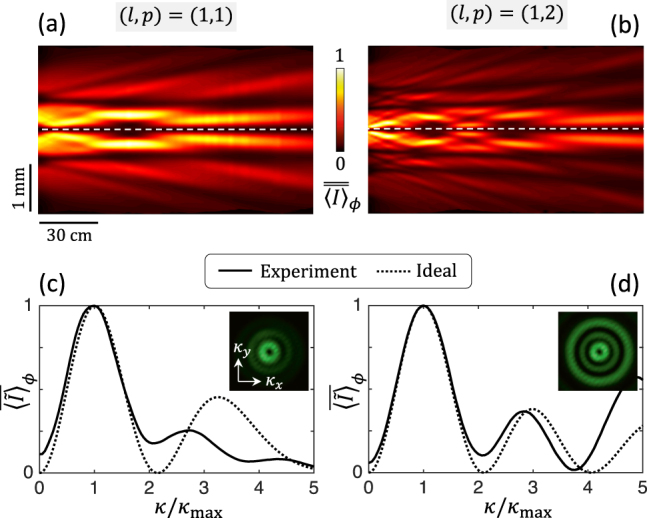
Experimental characterization of the propagation characteristics of modal vortex shapers with (*l*, *p*) = (1, 1) and (*l*, *p*) = (1, 2). (a) and (b) Reduced azimuth-average intensity distribution in the (*z*,*r*) plane. (c) and (d) Reduced azimuth-average far field intensity distribution and transverse intensity distributions (insets). The notations and the data recording protocols are identical to that of Figures 3 and 5.

The structural characterization of these high-order modal optical elements is shown in Figure 6, which follows the approach used for the characterization of the modal shapers for *p* = 0 and similar comments apply. In addition, the propagation analysis is summarized in Figure 7. Although the prescribed design is fairly validated both for the retardance and the azimuth and that the far field beams exhibiting *p* + 1 ring intensity profiles, the propagation-invariant intensity profile up to a radial stretching factor is poorly validated at a finite distance from the optical elements. This could be explained by pointing out a dynamic phase issue that does not appear when simply looking at the polarization vector modification from a uniform retarder as described by Eq. (2). Indeed, the output local Jones vector is defined up to a pure phase factor proportional to exp[*i*Φ_dyn_] where Φ_dyn_ is the dynamic phase accumulated as light passes through the modal beam shaper. This phase depends on the average refractive index $\bar{n}$ of the laser-structured artificial uniaxial medium, the thickness *L* of the structured material and the nature of the structure itself. As the fabrication process implies the control of the artificial retardance, yet without simultaneous control of the dynamic phase [11], we expect the modal shapers to be endowed with an *r*-dependent dynamic phase. Our experimental results suggest that this is especially detrimental for higher-order radial modes and further work is necessary to make the present approach a turnkey technology.

## 6 Discussion

Phase compensation strategies should be developed to fix the dynamic issue mentioned above. For instance, one could incorporate an extra geometric phase via a radially-varying perturbation d*ψ*(*r*) = −*σ*Φ_dyn_(*r*)/2 to the initial design dedicated to *σ*-polarized incident light, as theoretically proposed in [6]. However, this comes at the expense of breaking the symmetry between the two handedness for the incident circularly polarized Gaussian beam. Indeed, the fact that the correction is *σ*-dependent implies that the mono-modal character of the post-selected output field will be fine for *σ*-polarized incident beam while it will be worse than before correction for the −*σ* case. A recent work based on the use of metallic metasurfaces [10] proposed similar phase management scheme, which is however restricted to far-field experimental analysis. Another recent theoretical work dealing with dielectric metasurfaces [8] proposed an alternative management of both phase and amplitude that is not restricted to the use of circularly polarized incident fields, yet also restricted to operate for a single incident polarization state. Finally, recalling that the present artificial optical anisotropy results from laser-induced self-structured nanogratings in silica glass, one could implement a recently proposed bi-layer strategy based on the use of complementary subwavelength gratings having equal thickness and whose combined effects are predicted to lead to almost flat dynamic phase profile [21]. However, this comes at the expense of non-trivial fabrication difficulties since one deals here with self-structured subwavelength grating whose geometrical parameters are uneasy to control by nature. In other words, there is no easy way out of this problem.

## 7 Conclusions

Still, the present approach has the merit of being based on state-of-the-art technology that suits well any wavelength covering the visible and the near infrared domain and which is already commercially available for geometric phase retarders with uniform retardance. In addition, noting that macroscopic dimensions are readily achieved as shown here, we expect that further developments of the present proposal could lead to user-friendly optical components whenever enhanced modality is desirable. However, present day limitations regarding the spatial resolution based on laser-nanostructured silica glass prevents its use for integrated modal vortex beam shapers since controlled artificial optical properties inside a disk of a few tens of micrometers remains an open issue to date. The latter spatial resolution constraints of silica-based geometric phase vortex beam shapers have been overcome several years ago using photo-patterned liquid crystal polymer [22]. This technology can indeed provide sub-micron spatial resolution while preserving optical elements with centimeter-sized aperture, yet without spatially modulated retardance capabilities. To this aim, spontaneously formed liquid crystal topological defects endowed with both azimuthally varying optical axis orientation and radially varying retardance [23] is an option worth exploring in the framework of optical modality. Advances in nanofabrication provide another technological avenue for solving spatial resolution problems, as suggested by a very recent work in the case of metallic modal metasurfaces [10], yet being restricted to micro-optical elements and to piecewise designs. Further work in this direction could provide an extracavity laser beam shaping version of a recently proposed intracavity approach that relies on optical metasurfaces and for which the details of the modal filtering process at work remain to be fully understood [17].
